# A rapid, efficient, and economic device and method for the isolation and purification of mouse islet cells

**DOI:** 10.1371/journal.pone.0171618

**Published:** 2017-02-16

**Authors:** Yin Zongyi, Zou Funian, Li Hao, Cheng Ying, Zhang Jialin, Li Baifeng

**Affiliations:** 1 Department of Hepatobiliary Surgery and Organ Transplantation, the First Hospital of China Medical University, Shenyang, China; 2 National Key Lab. of General Surgery, the First Hospital of China Medical University, Shenyang, China; 3 Multiple Organ Transplantation Institute of the First Hospital of China Medical University, Shenyang, China; Tecnologico de Monterrey, MEXICO

## Abstract

Rapid, efficient, and economic method for the isolation and purification of islets has been pursued by numerous islet-related researchers. In this study, we compared the advantages and disadvantages of our developed patented method with those of commonly used conventional methods (Ficoll-400, 1077, and handpicking methods). Cell viability was assayed using Trypan blue, cell purity and yield were assayed using diphenylthiocarbazone, and islet function was assayed using acridine orange/ethidium bromide staining and enzyme-linked immunosorbent assay-glucose stimulation testing 4 days after cultivation. The results showed that our islet isolation and purification method required 12 ± 3 min, which was significantly shorter than the time required in Ficoll-400, 1077, and HPU groups (34 ± 3, 41 ± 4, and 30 ± 4 min, respectively; P < 0.05). There was no significant difference in islet viability among the four groups. The islet purity, function, yield, and cost of our method were superior to those of the Ficoll-400 and 1077 methods, but inferior to the handpicking method. However, the handpicking method may cause wrist injury and visual impairment in researchers during large-scale islet isolation (>1000 islets). In summary, the MCT method is a rapid, efficient, and economic method for isolating and purifying murine islet cell clumps. This method overcomes some of the shortcomings of conventional methods, showing a relatively higher quality and yield of islets within a shorter duration at a lower cost. Therefore, the current method provides researchers with an alternative option for islet isolation and should be widely generalized.

## Introduction

Diabetes is a chronic metabolic disease that affected 400 million patients worldwide in 2014, with an adult prevalence rate of 8.5%, and has become a major threat to human health [1]. Currently, islet transplantation is considered an ideal therapeutic approach for Type I diabetes, as it causes minor trauma and side effects in patients. However, the unavailability of a rapid, efficient, and economic method for isolating and purifying islets from donors remains an important limitation of studies related to islet transplantation and the molecular pathology of diabetes [2]. To overcome this issue, researchers have attempted to improve the isolation and purification method in order to establish a standard operating procedure [3, 4]. Islet isolation and purification procedures for large experimental animals, such as pigs, are relatively well-established [5]. Murine cells (rats and mice) have been widely used in various studies because of their advantages, such as high reproductive capacity, pure strains, good repeatability, short experiment time, and low cost. However, the fine anatomical structure and fragile tissues of murine (particularly mice) complicate islet isolation procedures. In this study, we introduce our improved patented device and method for islet isolation and purification using mice as an experimental animal model [6]. We also compared the advantages and disadvantages of our method with several conventional methods to achieve efficient, economic, and rapid isolation and purification of islets for use in research studies.

## Materials and methods

### Experimental materials

Male, specific pathogen-free C57b/6 mice, 8–12 weeks of age, were obtained from the Laboratory Animal Center of China Medical University. Collagenase-P was purchased from Roche, Ltd. (Switzerland). Ficoll-400 was purchased from Pharmacia Corporation (USA). Lymphocyte separation medium (LSM) (LTS1077) was purchased from Tian Jin Hao Yang Biological Manufacture Co., Ltd. (China). Hank’s balanced salt solution (HBSS) buffer, RPMI 1640 medium, and fetal bovine serum (FBS) were purchased from the Gibco (USA). The penicillin-streptomycin solution was from Sigma (USA). Diphenylthiocarbazone (DTZ) was purchased from Sinopharm Group Co., Ltd. (China). The acridine orange (AO)/ethidium bromide (EB) staining kit was purchased from BBI Healthcare Company (UK). The insulin enzyme-linked immunosorbent assay (ELISA) kit was purchased from Alpha Diagnostic Intl. Inc. (USA). The water bath was purchased from Shanghai Heng Industrial development Co., Ltd (China), and the centrifuge was purchased from Sanyo Electric Co., Ltd (China). Other consumables and reagents were purchased from Shenyang Boer Mei Biotechnology Co., Ltd. (China).

### Experimental methods

The study was approved by the Ethics Committee of China Medical University.

#### Preparation of isolating solution and digestive solution

Isolating solution was composed of 500-mL Hanks solution containing 10 mM of HBSS and 15 mM of CaCl_2_. The solution was filter-sterilized through a 0.22-μm filter and adjusted to pH 7.2–7.4 prior to storage at 4°C until use.

Digestive solution, in which the final concentration of collagenase-P was 1 mg/mL, was freshly prepared before use with the aforementioned isolating solution (pH 7.2–7.4).

#### Preparation of Ficoll-400 concentration gradient solution

Hundred grams of Ficoll-400 powder was added to 200-mL Hank’s solution. The solution was brought to 400 mL with Hank’s solution to prepare a 25% Ficoll solution, and 92, 80, and 44 mL of this solution was mixed with 8, 20, and 56 mL of Hank’s solution to prepare 23%, 20%, and 11% Ficoll solutions, respectively. These high-osmolality Ficoll-400-based discontinued density gradients were employed for islet purification, as previous described [7]. These solutions were filtered through a 0.22-μm filter membrane and adjusted to pH 7.2–7.4 prior to storage at 4°C.

#### Isolation of mouse pancreas

A mouse was anesthetized with 1.5% sevoflurane and fixed in the supine position. Its skin was disinfected with 75% ethanol followed by sterile laparotomy. The common bile duct (CBD) close to the duodenum was ligated for the retrograde puncture of the CBD, followed by a slow perfusion of 3 mL collagenase-P (pre-chilled at 4°C) to fully expand the pancreatic body and tail. The heart was excised to drain the blood and the pancreas was obtained by blunt isolation.

#### Improved method for isolation and purification of mouse islets

**Fig 1:**
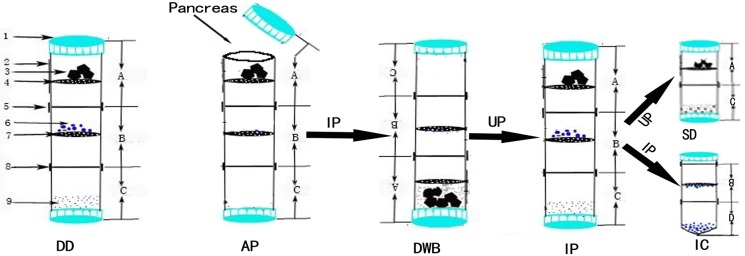
Device and flow chart of islet isolation and purification. (1. Centrifuge cap; 2. Matte surface of tube outer wall; 3. Pancreatic tissue; 4. Coarse sieve; 5 and 8. Detachable joints; 6. Islets; 7. Fine sieve; 9. Single-cell suspension; A. Upper tube section; B. Middle tube section; C and D. Lower tube section.); DD, device description; AP, acquisition of pancreas; IP, inverted position; DWB, digestion in water bath; UP, upright position; IP, islet purification; SD, second digestion; IC, islet collection.

Multi-layer centrifuge tube (MCT group) (Fig 1): The pancreas sample was transferred onto the first layer (Layer A) of a 50-mL multi-layer centrifuge tube (MCT) containing 2-mL pre-chilled collagenase-P (same concentration as the perfusion fluid). The MCT was incubated upside down in a water bath at 37°C for 8–20 min (different batches of collagenase-P result in fluctuation of digestion time, which must be optimized by the experimenter), followed by thorough vortexing in an inverted position for 30 s until the digestion solution appeared slurry. Next, the MCT was turned upright to carefully remove and transfer the fine sieve layer to a capped V-bottom centrifuge tube. The fine sieve layer was thoroughly rinsed with phosphate-buffered saline (pre-chilled at 4°C) and centrifuged with washing twice at 1000 rpm/min (×200*g*) for 2 min. Next, the supernatant was discarded while the pellet was suspended in RPMI 1640 medium (supplemented with 10% FBS) and transferred into a 37°C incubator until use. After removing the fine sieve layer (Layer B), the joining tubes were joined together and incubated upright in a refrigerator or on ice bath at 4°C. If the fine sieve layer yields fewer islets and microscopic inspection reveals incomplete digestion and the removal of islet follicular cells, the above procedure can be repeated for a second digestion. The entire procedure was carried out under aseptic conditions. The time required for digestion in the water bath, until islets were obtained and all reagents and consumables were used, and human resource demands were recorded.

#### Conventional isolation and purification of mouse islets

Ficoll-400 density gradient centrifugation (Ficoll-400 group): The aforementioned intact pancreas was transferred into a 50-mL centrifuge tube containing 2-mL pre-chilled collagenase-P (same concentration as the perfusion fluid) and digested in a water bath for 11–20 min at 37°C. Next, the centrifuge tube was vortexed thoroughly for 30 s until the digestive solution appeared cloudy, followed by the addition of 10-mL Hank’s solution supplemented with 10% newborn bovine serum (NBS) (pre-chilled at 4°C) to terminate the collagenase digestion. The solution was filtered through a 600-μm filter membrane, followed by centrifugation with washing twice at 1000 r/min for 2 min. The supernatant was discarded and the pellet was thoroughly suspended in 5-mL 25% Ficoll-400 solution. Subsequently, 2 mL each of 23%, 20%, and 11% Ficoll-400 solutions were successively and gently added, followed by centrifugation at 1800 r/min and 4°C for 15 min to collect the cell layers on the 20–11% interface (most islet cell clumps were deposited on this layer) and the 23–20% interface (small proportion of islet cell clumps). The islet cell clumps were centrifuged with washing twice with Hank’s solution containing 10% NBS at 1000 r/min for 2 min. The pellet was suspended in RPMI 1640 medium supplemented with 10% FBS and transferred to a 37°C incubator until use. The time required for digestion in the water bath until islets were obtained, as well as all reagents and consumables used and human resource demands, were recorded.

LSM (1077) density gradient centrifugation (1077 group): The same digestion method used for the Ficoll-400 group was performed. The solution was filtered through a 600-μm filter membrane followed by centrifugation with washing twice. The supernatant was discarded and the tube was drained in an inverted position on absorbent paper to remove trace supernatant on the tube wall (note: care should be taken to not perturb the pellet). To the centrifuge tube in the upright position, 3 mL of 1077 was added to thoroughly suspend the pellet by vortexing, after which 2 mL of 1077 was added through the tube wall to flush residual tissues on the tube wall. The suspension was carefully mixed by pipetting. This solution was mixed with 5-mL serum-free RPMI 1640 medium at 1 mL/10 s. The centrifuge tube was carefully transferred and centrifuged at 4°C and 2500 r/min for 20 min. Islet cell clumps at the interface between the 1077 and medium were obtained and mixed with 5-mL Hank’s solution supplemented with 10% NBS, followed by centrifugation with washing twice at 1000 r/min for 2 min to collect the pellet. The pellet was then suspended in RPMI 1640 medium supplemented with 10% FBS and incubated at 37°C until use. The time required for digestion in the water bath until islets were obtained, as well as all reagents and consumables used and human resource demands, were recorded.

Hand picking up (HPU group): The digestion method used for the Ficoll-400 group was performed and the solution was filtered through a 600-μm filter membrane followed by centrifugation with washing two times. Next, the pellet was re-suspended in Hank’s solution supplemented with 10% NBS (pre-chilled at 4°C) in a 10-cm culture dish. Because islets have a significantly distinct refractivity, morphology, and size from other pancreatic tissues, they can be hand-picked under a stereomicroscope using a capillary glass tube with an inner diameter of 500 μm (for large islets) or 200 μm (for small islets), or a 10-μL pipette tip. The resulting islets were centrifuged and washed twice with 10% Hank’s solution and suspended in RPMI 1640 medium supplemented with 10% FBS, followed by incubation at 37°C in an incubator until use. The time required for digestion in the water bath until islets were obtained, as well as all reagents and consumables used and human resource demands, were recorded.

#### Viability, yield, and functional assays of islet cell clumps

The viability of islet cells was determined using Trypan blue, which stains dead cells blue while viable cells remain unstained. The islet cells were added to 0.1 mL 0.4% Trypan blue (4% Trypan blue stock solution: 4 g Trypan blue was weighed and added to a small amount of distilled water, H_2_O with grinding. The solution was then brought to 100 mL with ddH_2_O, filtered through a filter membrane, and stored at 4°C until use. The stock solution was diluted to 0.4% using Hank’s solution before use). The islets were specifically stained with 0.1 g/L of DTZ to observe their morphology and to calculate yield and purity (purity = number of cell clumps stained with DTZ/total number of cell clumps × 100%). Fifty isolated and purified islet cell clumps from each group were inoculated into a 24-well plate and cultivated in RPMI 1640 medium supplemented with 10% FBS at 37°C; the medium was changed each day. On the fourth day of cultivation, AO/EB was used to determine the viability of the cell clumps, while insulin secretion under high-glucose (16.7 mM) and low-glucose (2.8 mM) stimulation were assessed by ELISA.

### Statistical analyses

Statistical analysis was carried out using Graphpad Prism 7 (GraphPad, Inc., La Jolla, CA, USA). Groups were compared and continuous variables were tested by analysis of variance, while binary variables were tested with the *t*-test. P < 0.05 indicated a significant difference.

## Results

### Analysis of process duration

We recorded the time required to isolate and purify the islet cells for each of the four experimental groups. We found that the duration of the MCT group was 12 ± 3 min, while the durations of the Ficoll-400 group, 1077 group, and HPU group were 34 ± 3, 41 ± 4, and 30 ± 4 min, respectively. The differences between the MCT group and other groups were significant (P < 0.05) (Table 1).

**Table 1:** Comparison of parameters of the four methods for the isolation and purification of islet cell clumps (6 experiments/group).

Parameters	MCT group	Ficoll-400 group	1077 group	HPU group
**Duration (±SD, min)**	12 (3)	34 (3)*	41 (4)*	30 (4)*
**Islet viability**	98.3%	96.5%	96.9%	99.0%
**Islet purity**	90.4%	87.8%	89.1%	98.6%*
**Insulin release index (±SD)**	4.5 (0.2)	3.2 (0.4)*	3.5 (0.3)*	4.7 (0.1)
**Number of islet clumps (±SD, clumps/mouse)**	144 (21)	149 (32)	157 (36)	223 (27)**
**Recovered islet count per duration (/min)**	12	4.4**	3.8**	7.4*
QIRRPTP	181 (13)	212 (36)*	194 (25)	238 (31)*
**Cost/100 islet clumps (RMB/Yuan)**	40	70*	70*	40
**Special reagents/equipment**	centrifuge tube, strainer mesh	Ficoll-400	1077 separation solution	Sterile stereomicroscope

Note:

* indicates P < 0.05;

** indicates P < 0.01.

QIRRPTP, the quantity of islets recovered to the required per transplantation procedure.

### Islet viability, purity, and function assays

**Fig 2:**
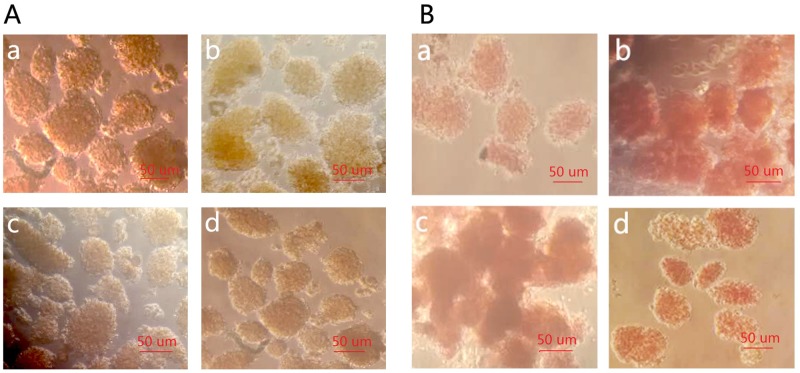
Islet purity and viability assays in each group (×20). (A: Result of Trypan blue staining and de-staining, B: Result of DTZ staining, a–d: MCT group, Ficoll-400 group, 1077 group, and HPU group, respectively).

To observe the outcomes of islet isolation and purification in each group (Table 1), we used Trypan blue to stain dead islet cells and calculate islet viability. The results showed that in the MTC and HPU groups, very few cells were stained blue with islet viability greater than 98%, while islet viabilities in the Ficoll-400 and 1077 groups were 96.5% and 96.9%, respectively. There was no significant difference among groups (Fig 2A). Further, we used DTZ to determine islet purity and found that the MCT, Ficoll-400, and 1077 groups yielded similar purity of up to approximately 90%, while the HPU group yielded up to more than 98% purity, showing a significant difference compared to the MCT group (P < 0.05) (Fig 2B).

With regards to the number of islets obtained from each mouse, for the MCT group, Ficoll-400 group, and 1077 group, close to 150 islet cell clumps were obtained, while for the HPU group, approximately 220 islet cell clumps were obtained, showing a significant difference from the MCT group (164 ± 21 cell clumps) (P < 0.05).

**Fig 3:**
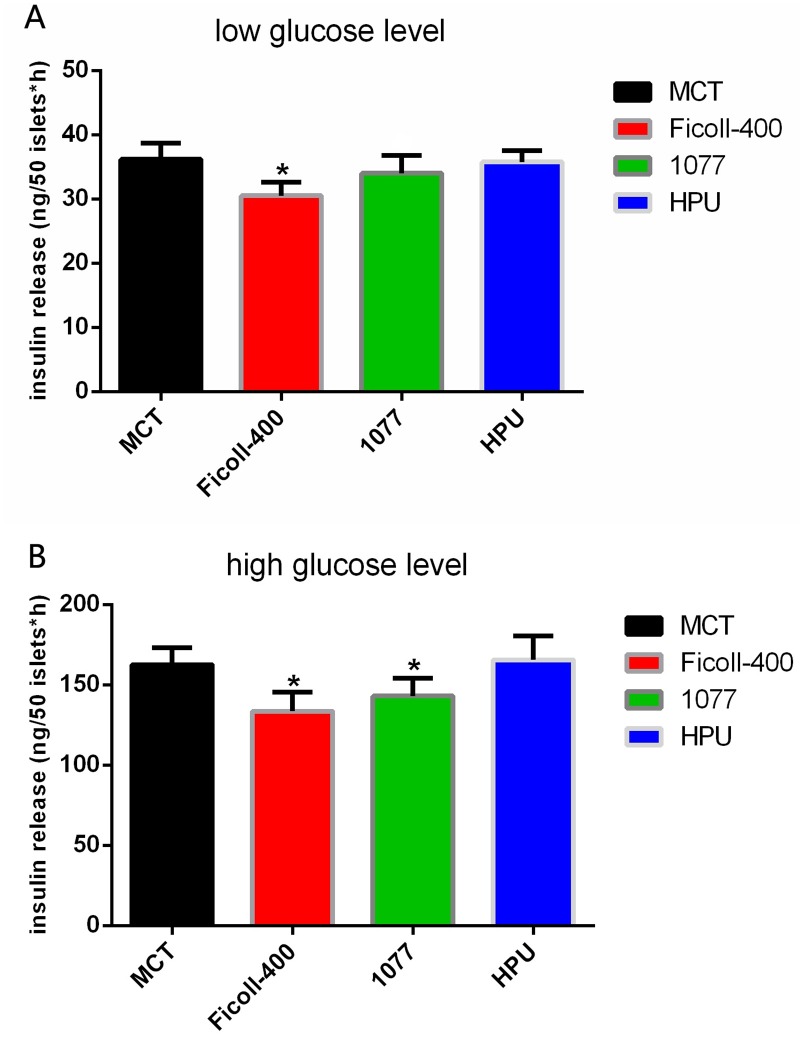
Insulin secretion stimulated by different glucose levels in each group.

**Fig 4:**
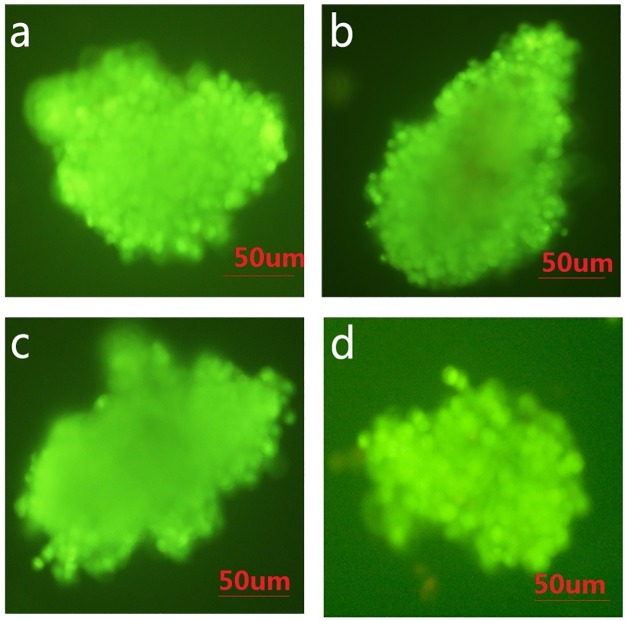
Growth of islets in each group after 4 days of cultivation (×40). (AO/EB staining, a–d: MCT group, Ficoll-400 group, 1077 group, and HPU group, respectively).

Next, we performed functional assays on the islets in each group on the 4^th^ day of cultivation (Fig 3). Insulin secretion in the low-glucose subgroup (2.8 mM) was 30–40 ng for all four groups; the difference between the Ficoll-400 group (31.2 ng) and MTC group (37.5 ng) was significant (P < 0.05). Insulin secretion in the high-glucose subgroup (16.7 mM) was 140–170 ng for all four groups; the differences between the Ficoll-400 group (137.8 ng) and 1077 group (143.4 ng) with the MCT group (162.5 ng) were significant (P < 0.05). The insulin index of the MTC group (4.5 ± 0.2) was significantly higher than that of the Ficoll-400 group (3.2 ± 0.4) and 1077 group (3.5 ± 0.3) and similar to that of the HPU group (4.7 ± 0.1). Additionally, the AO/EB staining results revealed that a small proportion of cells in the islet cell clumps of the Ficoll-400 and 1077 groups appeared apoptotic, whereas the islet cell clumps in the MCT group and HPU group grew well (Fig 4).

Furthermore, we analyzed the recovered islet count per duration in each group. We found that this parameter in the MCT group is 12 islets/min, which is higher than that in the Ficoll-400 group (4.4 islets/min), 1077 group (3.8 islets/min), or HPU group (7.4 islets/min). We also compared the quantity of islet cells recovered in each transplantation procedure. We found that the quantity of islets recovered in the MCT group (181 ± 13) was significantly less than that in the Ficoll-400 group (212 ± 36) and in the HPU group (220 ± 31). There was no notable difference between the MCT group and 1077 group (Table 1).

### Cost analysis

The costs of the Ficoll-400 group and 1077 group (70 Yuan/100 islets) were significantly higher than those of the MCT and HPU groups (40 Yuan/100 islets), as these methods required additional density gradient centrifugation solution (Ficoll-400 or LSM 1077) (P < 0.05, Table 1).

## Discussion

Islet transplantation is currently considered the only therapeutic approach for type 1 diabetes, and thus, an efficient, economic, and rapid isolation of islets is necessary for effective therapy. Over the past few decades, the isolation and purification of islets were carried out by tissue mincing and digestion, followed by mechanical grinding and digestion; however, both the yield and quality of the resulting islets needs to be improved [2, 5, 8].

Gotoh *et al*. [9] established a method for perfusing CBD with collagenase to digest the pancreas from the inside out, followed by Ficoll density gradient centrifugation. This method overcomes the limitation of low islet yields of previous methods, but the tedious preparation of Ficoll-400 density gradient solution prior to isolation and purification, as well as the toxic effects of Ficoll-400 solution on islets, affect the purity and quality of the resulting islets.

In 2011, Zmuda *et al*. [10] proposed replacing Ficoll-400 with human LSM (1077) as the density gradient solution as well as improving the corresponding procedures. The 1077 solution required for this method can be purchased directly without the need for researchers to prepare the density gradient solution. Additionally, the 1077 solution had no significant toxic effect on islet cells, further overcoming the multiple shortcomings of the Ficoll-400 method, and showed comparable purity and yield of islets to the Ficoll-400 method. However, similarly to the Ficoll-400 method, the 1077 method also exhibits low islet yields (small islets settle to the bottom of the tube), shows difficulties in determining the centrifugal density, and has higher experimental costs. To address these challenges, O'Dowd *et al*. [11, 12] developed a method for hand-picking islets under a stereomicroscope. The method is based on the distinct refractivity and staining intensity of islets compared to other pancreatic tissues under the stereomicroscope, and thus islets can be picked using a pipette and 10-μL tip. This method results in nearly 100% islet purity with a greater than 1.5-fold higher viability (>98%) and yield than the two methods described above (nearly all islets, both small and large, were isolated), making it an ideal graft material. However, this method also shows some limitations. It is only applicable in experiments that require a small number of islets (<1000 islets), as the hand-picking procedure is a major challenge to the physical and mental strength of operators, particularly the repeated pipetting, which easily leads to fatigue of the wrists and even tendinopathy. In addition, a long-term microscope session also causes visual impairment, affecting the health of the experimenters. Although numerous automated cell isolation instruments have been introduced, they are mainly used for the clinical isolation and purification of islets in human islet transplantation (>100,000 islets), are costly, and require operations personnel training. Additionally, the HPU method requires researchers to pick the islets individually, greatly prolonging the duration of isolation and purification, particularly for novice researchers. Furthermore, a typical laboratory is not equipped with a sterile stereomicroscope, greatly limiting the use of this technique.

We proposed a novel islet isolation and purification method based on an experimental device developed by our group, incorporating the advantages and overcoming the disadvantages of conventional methods. Our method has the following advantages compared to other methods:

First, it is rapid. The isolation of islets using our device is quicker than other methods because: 1) it requires fewer reagent-prep stages, one just needs to buy a developed multi-layer centrifuge tube (1–3 pancreatic samples per tube), and there is no need to buy and prepare density gradient centrifugation solutions, like Ficoll-400 or 1077-based purification solutions. 2) Its operating protocol is faster than that of the traditional gold-standard methods. All the procedures of digesting and purifying islets with our method are performed in one multi-layer centrifuge tube, with just one 5-min centrifugation. By contrast, it is necessary for both Ficoll-400 and 1077 groups to have at least three centrifugations and at least three tube-to-tube removals, both of which will take at least 30 min. In addition, isolating islets with the HPU method also takes about 30 min in our practice.

Second, it is efficient. Although there is no significant difference between our method and that of the Ficoll-400/1077 groups in the viability and purity of recovered islets, the recovered islet count per duration and insulin release index is notably higher in our method than in the Ficoll-400/1077 groups. Additionally, the quantity of islets recovered per transplantation procedure in our method is less than that in other groups. The possible reasons are that the average size of islets in our method is bigger than that in other groups, and the digestive process of our method involves less human intervention, reducing the risk of contamination and toxicity.

Third, it is economical. Apart from the cost of the developed tube, there is no need to pay for Ficoll-400 powder, 1077 separation solution, a sterile stereomicroscope, and extra centrifugation tubes for islet purification. The price of the developed tube is similar to a normal centrifugation tube (2 RMB/YUAN), but is far from the cost of Ficoll-400 powder (150 RMB/YUAN), 1077 separation solution (120 RMB/YUAN), and a sterile stereomicroscope (100,000 RMB/YUAN). Moreover, it is feasible that if the fine sieve layer yields fewer islets and microscopic inspection reveals islet follicular cells were incompletely digested, the digested procedure can be repeated for a second digestion without adding extra collagenase-P. Because unlike other methods, we do not neutralize the un-digested collagenase-P in digestive solution when we isolate islets from the digestive solution. Because of these reasons, the cost per 100 islet clumps in our method is less than that in other methods (P < 0.05). Additionally, compared with the handpicking procedure, our method does not challenge the physical and mental strength of operators. These challenges include hundreds to thousands of repeated pipetting movements, which easily leads to fatigue of the wrists and even tendinopathy, and a long-term microscope session, which causes visual impairment, affecting the health of the experimenters. Our method is particularly suitable for large islet isolation (>1000 islets). Together, our method not only decreases the cost of islet-related experiments, but also greatly reduces human resources and time.

Notably, our method requires the operator to patiently and meticulously configure the digestion time, as over-digestion will result in a lower islet yield and viability, whereas incomplete digestion will lead to the blockage of excessive islets on the upper sieve, thus reducing yield and purity. The optimal concentration of collagenase-P in this study was approximately 1 mg/mL, which may fluctuate in different batches. It is recommended to optimize the digestion time from 8 to 20 min every minute. The experimenters also can constantly adjust the experimental time interval from both ends to the middle. The freshly prepared collagenase solution should be placed on ice and used within 1 h. A fully expanded pancreas is a prerequisite for complete digestion of the pancreas, particularly the tail of the pancreas. The experimenters should attempt to maintain the time before incubation in a water bath and after perfusion. The simultaneous digestion of multiple pancreases should be carried out in separate centrifuge tubes: it is not recommended to place multiple pancreases into the same centrifuge tube. Thoroughly vortexing the centrifuge tube (40 times/10 s for 30 s) after digestion can improve islet yield.

In summary, the MCT method is a rapid, efficient, and economic method for isolating and purifying murine islet cell clumps. This method overcomes some of the shortcomings of conventional methods, showing adequate quality and yield of islets within a shorter duration at a lower cost. Therefore, the current method provides researchers with an alternative option for islet isolation, and should be widely generalized.

## Abbreviations

AO/EB: acridine orange/ethidium bromide
CBD: common bile duc
DTZ: diphenylthiocarbazone
ELISA: enzyme-linked immunosorbent assay
FBS: fetal bovine serum
HBSS: hank’s balanced salt solution
HPU: hand picking up
LSM: lymphocyte separation medium
MCT: multi-layer centrifuge tube
NBS: newborn bovine serum
